# Exploring the interactions of the RAS family in the human protein network and their potential implications in RAS-directed therapies

**DOI:** 10.18632/oncotarget.12416

**Published:** 2016-10-03

**Authors:** Anibal Bueno, Ian Morilla, Diego Diez, Aurelio A. Moya-Garcia, José Lozano, Juan A.G. Ranea

**Affiliations:** ^1^ Departmento de Biología Molecular y Bioquímica, Universidad de Málaga, Málaga 29071, Spain; ^2^ Institute of Molecular Life Sciences, University of Zurich, Zurich CH-8057, Switzerland; ^3^ Quantitative Immunology Research Unit, World Premier International Immunology Frontier Research Center, Osaka University, Osaka 565-0871, Japan; ^4^ Institute of Structural and Molecular Biology, University College London, London WC1E 6BT, UK; ^5^ Instituto de Investigación Biomédica de Málaga (IBIMA), Hospital Universitario Virgen de la Victoria, Málaga 29010, Spain; ^6^ CIBER de Enfermedades Raras, Madrid 28029, Spain

**Keywords:** Ras, network, cancer, signaling, therapy

## Abstract

RAS proteins are the founding members of the RAS superfamily of GTPases. They are involved in key signaling pathways regulating essential cellular functions such as cell growth and differentiation. As a result, their deregulation by inactivating mutations often results in aberrant cell proliferation and cancer. With the exception of the relatively well-known KRAS, HRAS and NRAS proteins, little is known about how the interactions of the other RAS human paralogs affect cancer evolution and response to treatment. In this study we performed a comprehensive analysis of the relationship between the phylogeny of RAS proteins and their location in the protein interaction network. This analysis was integrated with the structural analysis of conserved positions in available 3D structures of RAS complexes. Our results show that many RAS proteins with divergent sequences are found close together in the human interactome. We found specific conserved amino acid positions in this group that map to the binding sites of RAS with many of their signaling effectors, suggesting that these pairs could share interacting partners. These results underscore the potential relevance of cross-talking in the RAS signaling network, which should be taken into account when considering the inhibitory activity of drugs targeting specific RAS oncoproteins. This study broadens our understanding of the human RAS signaling network and stresses the importance of considering its potential *cross-talk* in future therapies.

## INTRODUCTION

The RAS protein family is a set of small GTPases that function as binary switches by alternating their activation state from GTP-bound (active) to GDP-bound (inactive). In higher eukaryotes these proteins are involved in signal transduction pathways controlling a diverse array of essential cellular functions, such as growth, differentiation and survival [1]. In the human genome, the *RAS* family includes a large number of related genes (paralogs). However, with the exception of a few well-studied protein models, the precise functions of the thirty-five human RAS paralogs and their relation in terms of sequence conservation, gene expression and protein-protein interactions remain poorly understood [2].

Of clinical relevance, up to 30% of all human tumors present oncogenic mutations in members of the prototypical RAS family which often contribute to tumor pathogenesis by overactivating the Raf/MEK/ERK pathway [3, 4]. *KRAS* is the most frequently mutated *RAS* gene, accounting for up to 20% of all tumors. This is in marked contrast to *NRAS* and *HRAS* genes, found to be mutated in 5% and 3% of all tumors analyzed, respectively. In particular, *KRAS* mutations are predominant in pancreatic tumors, with an incidence as high as 90% (all data obtained from the Catalog Of Somatic Mutations In Cancer, COSMIC, http://cancer.sanger.ac.uk/cosmic [5]). Oncogenic RAS mutations are predominantly found in residues G12, G13 and Q61, impairing the intrinsic GTP hydrolysis and therefore, rendering RAS proteins in a permanent GTP-bound, active state [6]. In addition to cancer, mutations in *HRAS* and *KRAS* genes have been associated with the Costello and Noonan syndromes, respectively [7, 8]. Notably, other members of this gene family are not significantly mutated in cancer and only in some cases, overexpression of *RAS*-related genes has been associated to certain types of tumors, i.e. RALA and RALB are overexpressed in melanoma and non-small cell lung cancer (NSCLC), with RALA having a predominant role in tumor growth and RALB in its metastatic potential [9, 10].

**Figure 1:**
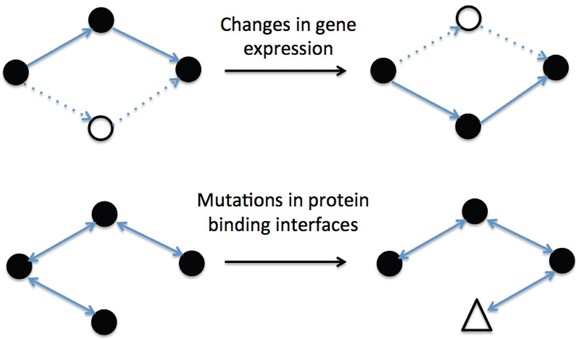
Examples of different biological mechanisms for changing interactions network topology The graphic on the top, where black nodes represent expressed proteins and solid lines active interactions, shows the effect of changing gene expression. Meanwhile, the graphic on the bottom represents the effect of a rewiring process induced by mutations (triangle) in protein binding interfaces.

As shown in Figure 1, the human RAS protein network can change its topology through two basic mechanisms: i) changing the nodes present in the network (i.e. changes in gene expression); and ii) rewiring the connections between nodes (i.e. mutations in the protein-protein binding interfaces). Although activated, wild-type Ras GTPases bind their downstream effectors with high affinity, the switchable nature of their activation mechanism (GTP/GDP exchange) can result in relatively transient protein-protein interactions (PPIs), which are susceptible to rewiring [11–16].

While the three prototypical RAS proteins had been extensively characterized, much less is known about the remaning RAS paralogs in either healthy or tumor tissues. In this work, we study the relationship between phylogenetic distances of all RAS paralogs and their associations in the human protein interaction network. To this end, we implement a comparative sequence analysis to find conserved amino acid positions between divergent RAS-protein pairs that preserve protein interaction network proximities in the human interactome. We hypothesize that these positions may help maintain functionally important protein interactions common to both paralogs resulting in close network proximity. These positions are then mapped onto different RAS complexes using their 3D structural information in order to determine their connection to RAS protein binding sites.

The results we show here add a new perspective to the generally accepted idea that the interactions between paralogous proteins diverge with their sequence [17–19] and shed some light on the largely unknown role of the human RAS interaction network. Furthermore, our findings broaden the current perspective on the putative role of paralogous genes in the development and adaptation of functional and pathological RAS signaling networks. In addition, important conclusions can be drawn out of the conserved positions in Divergent but Interacting RAS Pairs (DIRP) regarding their potential functional relevance for the design and development of new Ras inhibitors.

## RESULTS

### Phylogenetic and network distance relationships of human *RAS* paralogs

**Figure 2:**
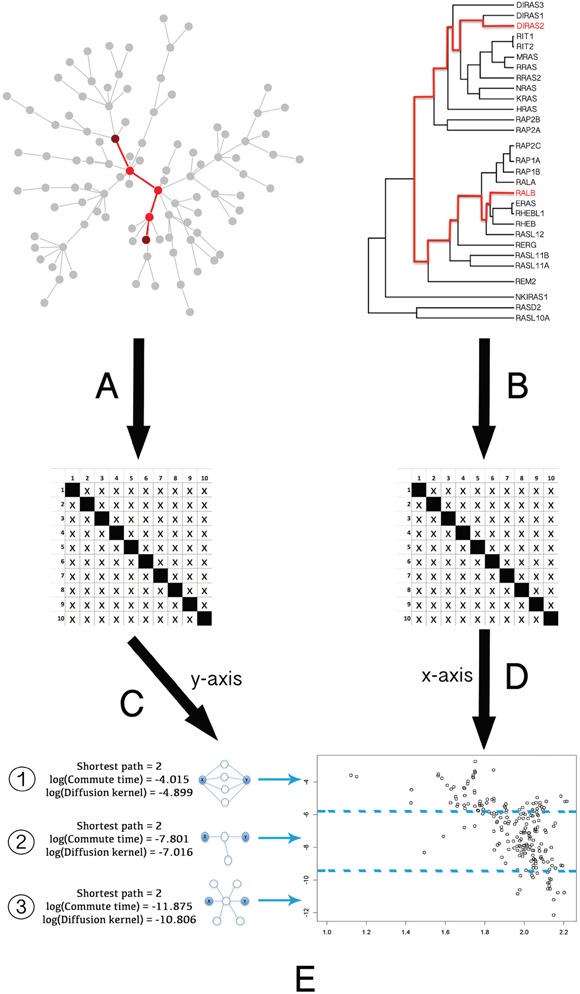
General pipeline of the Ras protein pairs phylogenetic and network distance measurements and comparison **A.** Pairwise distance calculation in the PPI graph, expressed as a matrix. **B.** Pairwise phylogenetic distance calculation in the tree, expressed as a matrix. **C.** Logarithmical transformation to normalize network distances between proteins. **D.** Exponential transformation to normalize phylogenetic distances between proteins. **E.** Graphical representation of both the proteins phylogenetic and network distances. As we can see in the left side of E, distance measures based on kernels (e.g. DK or CT), compared to shortest path calculation (minimum number of edges connecting two given nodes), are able to distinguish the level of association between two Ras nodes connected through different topologies: 1) highly connected nodes; 2) low connected; 3) nonspecifically connected. This result demonstrates that kernel similarity metric is one of the better measures to deal with the kind of artifacts produced by highly connected network hubs (see section 1 in Supplementary material).

**Figure 3:**
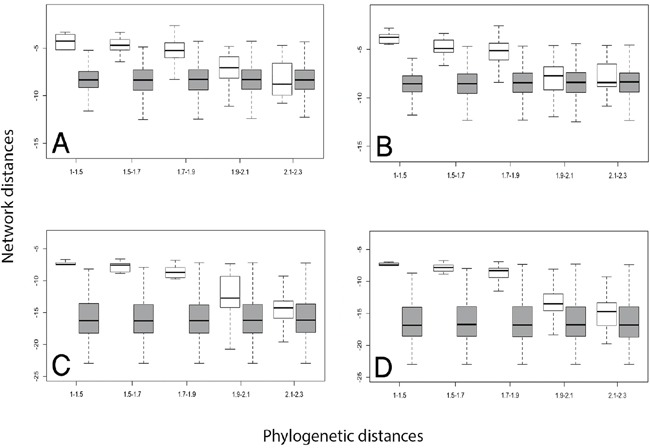
Distributions of network distance values between protein pairs in different phylogenetic distance bins Real (white boxes) and random (dark boxes) normalized distributions of the network distances between Ras protein pairs (y-axis), divided into bins corresponding to rising ranges of normalized phylogenetic distances (x-axis). Network distances were calculated applying CT (panels **A** and **B**) and DK (panels **C** and **D**) algorithms for the STRING Experimental (panels A and C) and the PINA (panels B and D) PPI graph datasets.

To analyze the relationship between the phylogeny of RAS proteins and their location in the protein-protein interaction (PPI) network, we compared the network and phylogenetic distances for human RAS paralogous pairs (see Figure 2 and Methods). RAS paralogs tended to be closely associated in the interactome when they were phylogenetically close and to increase their distance as they diverged. We observed the same pattern regardless of the PPI dataset and the network distance measure used (Figure 3). This pattern was absent in the random model (see Methods). As seen in Figure 3A-3D, network distances of the most divergent pairs resembled a random distribution, while phylogenetically close pairs had a very distinct network distance distribution.

The inverse correlation between sequence similarity and the phylogenetic distance of Ras protein pairs is consistent with an evolutionary model by which recently duplicated genes share the same context of interactions. Thus, as sequences diverge by accumulation of mutations, they move away from each other in the interactome. However, our results show that some of the distant duplicated genes keep the same protein-protein interaction context, suggesting that there is more to this model.

### Identification of divergent Ras paralog pairs located close in the PPI network

**Figure 4:**
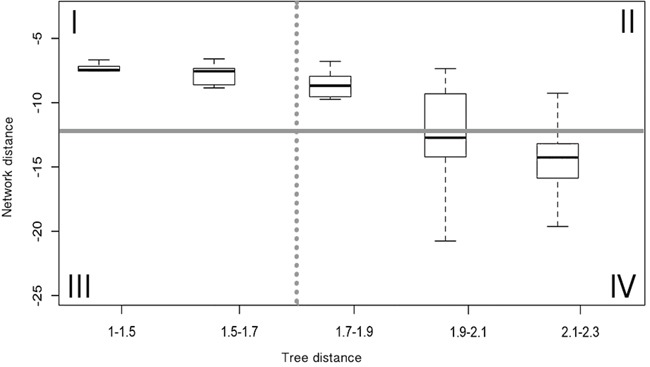
Distribution of the network vs phylogenetic distance values and established thresholds Example of the comparison between normalized phylogenetic distances and normalized network distances between protein pairs, applying DK algorithm to STRING Experimental dataset for obtaining network distances. The phylogenetic distance threshold corresponds to pairs with 45% sequence identity (dashed line) and network closeness threshold is established according to a p-value of 0.05 (solid line).

There is an inverse correlation between sequence conservation and the phylogenetic distance of Ras protein pairs. From this we can also conclude an inverse relationship between sequence conservation and network distance based on the results shown in Figure 3A-3D. An observation that suggests that conservation or variation of amino-acid positions would determine whether a pair of RAS proteins has the same or different neighbors in a PPI network. With the aim to identify amino-acid positions determinant of Ras proteins' location in the PPI network, we closely examined the relationship between the phylogenetic and network distance distributions of all Ras pairs. We distinguished four main panels in the phylogenetic vs. network distance plots based on two values used as boundaries, one for the network distance measures and another for the phylogenetic distances (panels I-IV in Figure 4): Panel I) Ras pairs close in the phylogenetic tree and in the PPI network graph, in this panel the general high conservation between sequences makes it difficult to distinguish those conserved positions responsible for the close network location observed in this set of pairs; Panel III) Ras pairs close in the tree and distant in the PPI network, this panel is empty, suggesting that a few mutations in recently duplicated *RAS* genes cannot produce a substantial change in their protein interaction contexts; Panel IV) Ras pairs distant in the tree and in the PPI network, in this panel IV the high divergence between sequences again makes it difficult to identify those variable positions directly responsible of the divergence in the interaction contexts of these pairs; finally, Panel II) Ras pairs distant in the tree but close in the PPI network, in this panel we find a set of divergent sequence pairs where it would be feasible to identify specific conserved positions related to their close location in the network. We refer to this set of pairs of paralogs as DIRP (Divergent but Interacting RAS Pairs).

In order to distinguish from meaningless random behavior, the DIRP dataset was selected out of all RAS pairs based on two statistical thresholds of significance: i) significant sequence divergence between proteins in the pair and ii) significant closeness in the protein interactome (Methods). The number of protein pairs that were finally included as DIRP is shown in Table 1, for each PPI network model and each network distance metric used.

**Table 1:** Number of protein pairs through all the selection process for obtaining the DIRP

	DK STRING Exp	CT STRING Exp	DK PINA	CT PINA
# initial pairs	351	351	435	435
#pairs after phylogenetic boundary	323	323	396	396
# DIRP	106	82	113	86
% pairs	30	23	26	20

The first row indicates the number of protein pairs that were initially analyzed in each system (algorithm and dataset used). The second row shows the number of pairs after applying the phylogenetic threshold for distant pairs (normalized phylogenetic distance ≥ 1.7). The third row contains the number of DIRP finally selected, after filtering by the normalized network distance threshold (p-value ≤ 0.05) established by means of random models and specified in Table S2 in Supplementary material. The last row indicates the percentages of DIRP over the total number of Ras pairs initially found.

We studied the relationship between network closeness and the similarity of interaction interfaces in the DIRP dataset by retrieving all the directly shared interacting partners for all pairs in the DIRP dataset, and comparing against an equivalent No-DIRP dataset (see Supplementary Figure S2). The DIRP pairs show a median of 3 shared interacting proteins per pair while in the No-DIRP dataset the median is zero. The results are practically the same if using Commute Time (CT) or Diffusion (DK) kernel similarity metrics. These results support a positive relationship between the number of shared interacting proteins (which bind to similar interfaces in Ras paralogs) and network closeness measured with kernel metrics. A detailed analysis of some DIRP pairs and their direct interactors (see section 3 in Supplementary material) shows that the majority of these shared physical interactions are cited in literature or annotated in functionally curated databases, although many of these interactions remain yet unpublished waiting for a functional study (Supplementary Table S5). The set of published shared interactions constitute a positive validation that support the cross-talk hypothesis between DIRP Ras paralogs.

### Searching for conserved positions in divergent but interacting RAS pairs (DIRPs)

**Figure 5:**
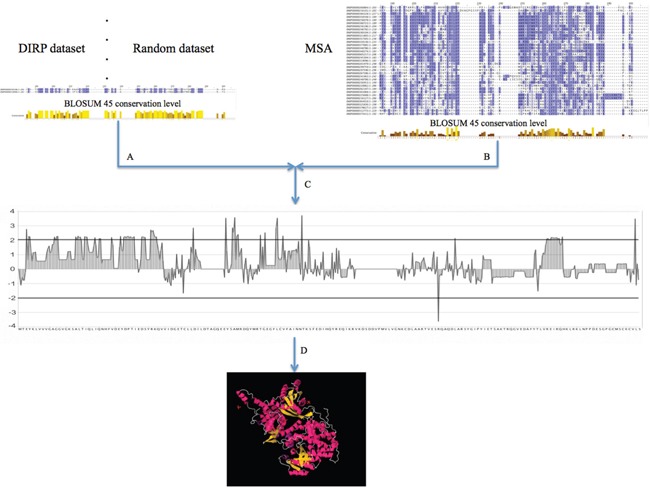
General pipeline for obtaining the set of DIRP specific positions and mapping them into Ras 3D complexes **A.** Position conservation measurement using the BLOSUM 45 matrix for the pairs selected as DIRP and for the randomly selected pairs (negative control). **B.** Position conservation measurement within the whole MSA. **C.** Differential position conservation (normalization) between both (the DIRP and random datasets) versus the MSA background. **D.** Selection of the significant DIRP specific positions and mapping on the different human Ras 3D binding complexes.

**Figure 6:**
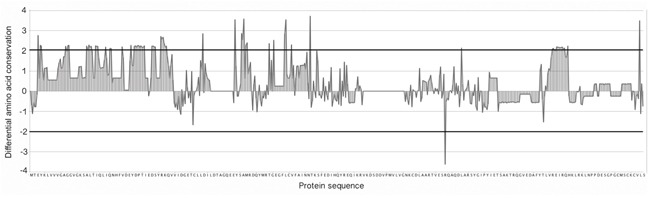
Differential amino acid conservation Differential position conservation between the DIRP dataset sequences and the general MSA (y-axis). Residues used as template correspond to the human HRas amino acid sequence (x-axis). A positive value in the difference of conservation indicates a position with higher level of conservation in the DIRP dataset than in the background dataset and a negative value indicates a position with a higher variability. Horizontal dark lines correspond to thresholds associated to p-values < 0.01.

In order to find the specific conserved positions within the DIRP set, all RAS protein sequences were aligned using a general multiple sequence alignment (MSA). Then, for each amino acid position, we normalized their conservation value in the positive (DIRP) and negative (random model) datasets by comparing it with the conservation of these same positions in the whole MSA background dataset (see Figure 5 and Methods). This normalization allowed us to identify positions significantly and specifically conserved in the DIRP dataset compared against both datasets (the random and the whole background MSA). With this approach we selected a total of twenty-two positions (p-value < 0.01, upper and lower thresholds in Figure 6) specific to the DIRP dataset. Twenty-one of these positions show a higher conservation in the DIRP dataset, while only one out of the twenty-two positions shows a higher variability (lower conservation) in the DIRP dataset (position R139 using HRas as reference in the alignment, see Supplementary Table S1). The absence of Ras protein pairs that are similar in sequence but separated from each other in the interactome (Panel III in Figure 4) contrasts with the abundance of highly divergent Ras pairs close in the network (Panel II in Figure 4). This suggests that a protein needs to accumulate many neutral and adaptive point mutations in order to get new interacting partners, whilst it can maintain it interaction context through a few key conserved positions.

### Relationship between the DIRP conserved positions and the Ras protein binding regions

**Figure 7:**
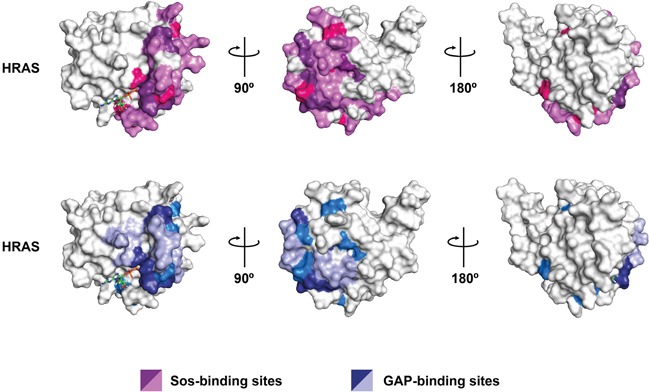
Spatial distribution of all DIRP in HRAS protein Relevant residues are positioned on a surface model for the 3D structure of the human HRAS paralog (pdb# 1aa9). Upper row: residues involved in the interaction of HRAS with the GEF Sos are in light purple while those DIRP involved in the interaction are in dark purple. DIRP not involved in the HRAS-Sos interaction are in pink. Lower row: residues involved in the interaction of HRAS with the GAP are in light blue while those DIRP involved in the interaction are in dark blue. DIRP not involved in the HRAS-GAP interaction are in marine blue. Note that both common and specific DIRP positions can be identified following this approach. For clarity, three rotating views are shown for each HRas protein.

In order to investigate the relationship between the DIRP specific positions and Ras protein binding sites, we collected twenty-eight RAS human complexes from the Protein Data Bank (PDB) [20] and clustered them into six structural groups (Methods). We then defined the binding regions between Ras and its partners based on the analysis of these structural groups (Table 2). Out of the 22 DIRP specific positions identified in the previous step, 15 (68%) are directly involved in one or more binding regions and are located in some of the functional regions identified in Ras proteins (Table 3, Table 4 and Figure 7). Another four are surrounded by two consecutive interacting positions in the amino acid sequence. Considering that these last positions may also be involved in Ras protein-protein interactions, we can conclude that 86% of the DIRP specific positions participate in the interactions of Ras with other proteins (Table 3). The remaining three were not related to any known interaction site in this analysis. These results indicate that DIRP specific positions are important to establish interactions between Ras and its partners and therefore their conservation can be an important factor in maintaining these phylogenetically distant Ras paralogs close in the interactome.

**Table 2:** DIRP specific positions mapping to binding sites in the human Ras complexes

Functional Group	Complexes	Description	Positions	Num	Ratio
RasGef	1LFD	Interaction of Ras with RalGDS	**G12**, **Y32**, **D33**, **P34**, **I36**, E37, D38, S39, Y40, Q61, E62, E63, **Y64**, S65, **A66**, M67	7/16	43.7%
	1NVU 1NVX 1NVW 1NVV	Feedback activation by Ras. GTP of the Ras-specific nucleotide exchange factor SOS	S17, **T20**, I21, **Q22**, I24, N26, H27, D30, E31, **Y32**, **D33**, **P34**, **I36**, E37, D38, Y40, K42, Q43, V44, **D54**, I55, D57, **A59**, **G60**, Q61, E63, **Y64,** S65, **A66**, M67, D69, Q70, **Y71**, R73, R102, R149	12/36	33.3%
	1XD2	Autoinhibition in the Ras activator Son of sevenless: ternary Ras:SOS:Ras*GDP complex	**Q22**, I24, N26, H27, **D33, P34**, **I36**, E37, D38, K42, Q43, V44, L56, E63, **Y64**, **A66**, M67, Q70, **Y71**, R149	7/20	35.0%
	1BKD	The structural basis of the activation of Ras by Sos: H-Ras with SOS-1	S17, I21, **Y32**, **P34**, Y40, **D54**, I55, D57, **A59, G60**, Q61, E63, **Y64**, S65, **A66**, M67, D69, Q70, **Y71**, R73, R102	8/21	38.1%
RapGef	3CF6	Epac2 in complex with a cyclic AMP analogue and RAP1B	S17, **T20**, I21, H27, **Y32**, **P34**, E37, Y40, **D54**, I55, L56, D57, **A59**, **G60**, Q61, **Y64**, **A66**, M67, D69, Q70, **Y71**, Q99	9/22	40.9%
RasGap	1WQ1	The Ras-RasGAP complex: structural basis for GTPase activation and its loss in oncogenic Ras mutants	A11, **G12**, G13, I21, **Y32**, **D33, P34**, **I36**, E37, D38, S39, Y40, **G60**, Q61, E62, E63, **Y64**, K88, D92	7/19	36.8%
Antobodies (Cancer supressors)	2UZI	Tumour prevention by a single antibody domain targeting the interaction of signal transduction proteins with RAS	I21, V29, **D33, P34**, **I36**, E37, D38, Y40, Q61, **Y64**	4/10	40.0%
	2VH5	HRAS(G12V) - ANTI-RAS FV (DISULFIDE FREE MUTANT) COMPLEX	I21, V29, **D33, P34**, **I36**, E37, D38, Y40, D57, Q 61, **Y64**	4/11	36.4%
	3DDC	Ras effector interaction between tumour suppressor NORE1A and Ras switch II	I24, Q25, **I36**, D38, Y40, **Y64**, M67	2/7	28.6%
Ras Binding Domain & PI3K	1HE8	Ras binding to its effector phosphoinositide 3-kinase gamma	I21, **D33**, **I36**, E37, D38, S39, Y40	2/7	28.6%
	3KUC 1GUA	Complex of Rap1A(E30D/K31E) GDP with RafRBD(A85K/N71R) Ras/Rap effector specificity determined by charge reversal	I21, **D33**, **I36**, E37, D38, S39, Y40, R41	2/8	25.0%
	3KUD	What makes Ras an efficient molecular switch: Ras-GDP interactions with mutants of Raf	I21, E37, D38, S39, Y40, R41	0/6	0.0%
	1K8R	Ras-Byr2RBD complex: structural basis for Ras effector recognition	**I36**, E37, D38, S39, Y40, R41, **D54**	2/7	28.6%
	1C1Y	c-Raf1 in complex with Rap1A and a GTP analogue	I21, **I36**, E37, D38, S39, Y40, R41	1/7	14.3%
Other cases	1ZC3 1ZC4	Ral-binding domain of Exo84 in complex with the active RalA	D47, G48, E49, T50, C51, L52, M67, G75, F78, V81, **F82**	1/11	9.1%
	2A9K 2A78	C3bot-NAD-RalA complex: Ral-A and Mono-ADP-ribosyltransferase C3 C3bot-RalA complex	**T20**, I21, **Q22**, L23, D69, **Y71**, M72, G75, L79, A83, **V103**, S106, D107, P110	4/14	28.6%
	2C5L	PLC epsilon Ras association domain with HRas	I24, Q25, **I36**, D38, S39, Y40, D47, S127, Q131, A134, Y141, I142, E143, D154, R161, R164, Q165	1/17	5.9%
	4DXA	Rap1 in complex with KRIT1	Q25, H27, **I36**, E37, D38, S39, Y40, Q43, M67	1/9	11.1%
	3T5G	Rheb in complex with PDE6D	T2, D57, G178, P179, G180	0/5	0.0%
	2BOV	recognition of an ADP-ribosylating Clostridium botulinum C3 exoenzyme by RalA GTPase	**I139**, P140, E143	1/3	33.3%
	1UAD	Interaction between RalA and Sec5, a subunit of the sec6/8 complex	**I36**, G48, E49, T50, C51	1/5	20.0%

From left to right: Functional group labels for clustered complexes; PDB Id codes of the Ras 3D complexes clustered with r.m.s. < 1.0; description of the Ras 3D complexes; positions involved in the binding site of the Ras complexes (those matching the DIRP specific positions are in bold); Number (Num.) and percentage (Ratio) of DIRP specific positions over all binding site positions. Positions numbering follows the human HRas protein sequence as reference.

**Table 3:** Ranked list of the DIRP specific positions based on their level of implication in Ras binding sites

Position	Number of matches	Complexes
I36	14	1NVU, 1XD2, 1LFD, 1WQ1, 2UZI, 2VHS, 3DDC, 1H8E, 3KUC, 1K8R, 1C1Y, 2C5L, 4DXA, 1UAD
Y64	9	1NVU, 1XD2, 1BKD, 1LFD, 3CF6, 1WQ1, ZUZI, 2VHS, 3DDC
D33	8	1LFD, 1NVU, 2UZI, 2VH5, 1HE8, 1XD2, 1WQ1, 3KUC
P34	8	1LFD, 1NVU, 2UZI, 2VH5, 1XD2, 1WQ1, 1BKD, 3CF6
Y32	5	1NVU, 1BKD, 1LFD, 3CF6, 1WQ1
A66	5	1NVU, 1XD2, 1BKD, 1LFD, 3CF6
Y71	5	1NVU, 1XD2, 1BKD, 3CF6, 1ZC3
D54	4	1NVU, 1BKD, 3CF6, 1K8R
G60	4	1NVU, 1BKD, 3CF6, 1WQ1
T20	3	1NVU, 3CF6, 2A9K
A59	3	1NVU, 1BKD, 3CF6
Q22	3	1NVU, 1XD2, 2A9K
G12	2	1LFD, 1WQ1
V103	1	2A9K
I139	1	2BOV
T35	0	Between interacting positions 34 & 36 in several complexes
R68	0	Between interacting positions 67 & 69 in several complexes
T58	0	Between interacting positions 57 & 59 in some complexes
F28	0	Between interacting positions 27 & 29 in some complexes
G77	0	
E153	0	
C186	0	

First column shows the amino acid position according to HRas sequence. Second column indicates the number of 3D complexes binding sites in which the position is directly involved. Third column contains PDB Id codes for the complexes that are related to each position or annotation of indirect relationships to binding sites.

**Table 4:** DIRP specific positions clustered in Ras functional regions

Functional Regions	Positions	Ratio
Switch I (Effectors binding site)	Y32, D33, P34, T35, I36	23%
Switch II	G60, Y64, A66, R68, Y71	23%
C-terminal hyper variable region	C186	5%
Nucleotide (GDP/GTP) binding site	G12, F28, T35, T58, A59, G60	27%
Innert regions	T20, Q22, D54, G77, V103, I139, E153	32%

First column lists the different functional region in Ras proteins. Second column indicates the DIRP amino acid position according to HRas sequence. Third column shows the percentage (Ratio) of DIRP specific positions in each functional region.

**Figure 8:**
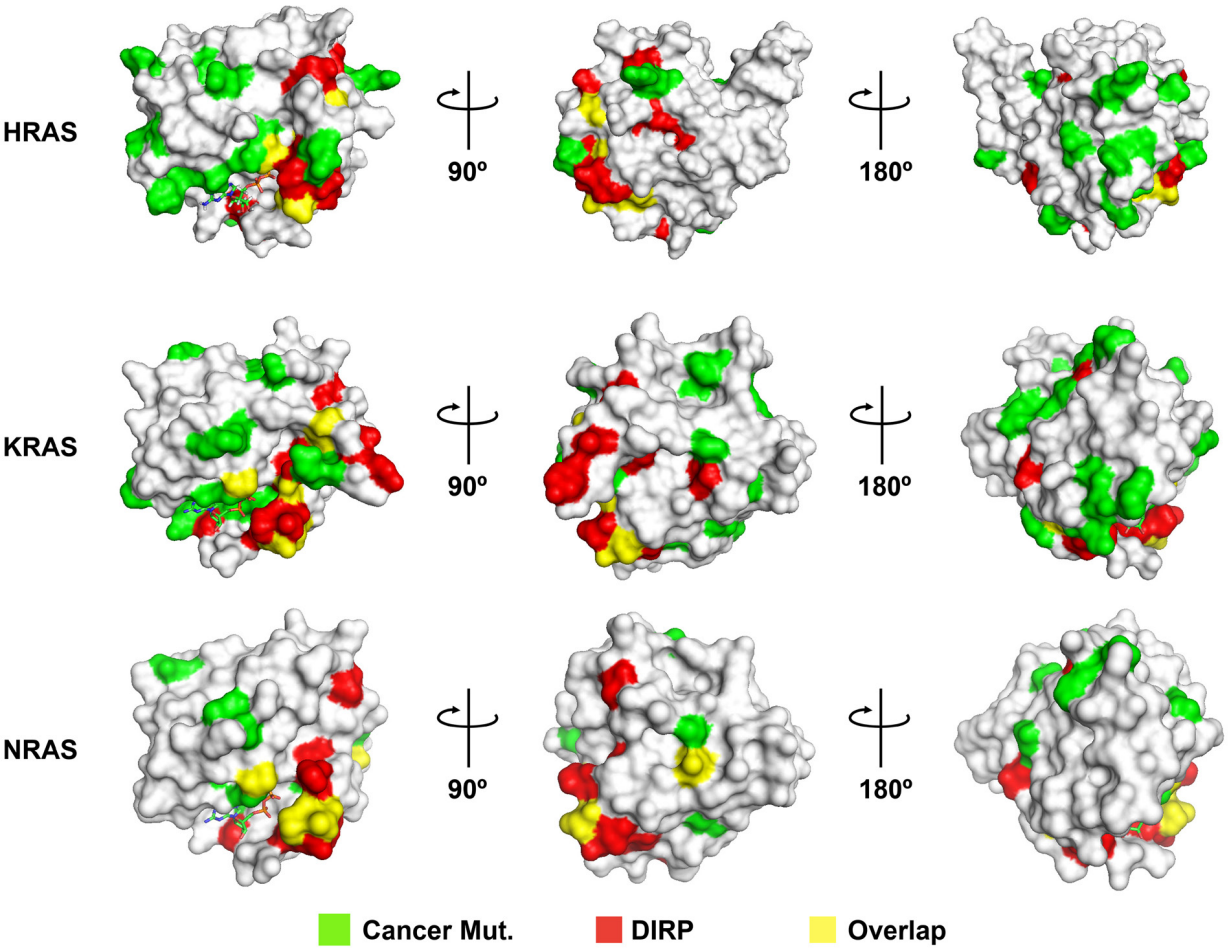
DIRP overlap with or are positioned near to residues frequently mutated in cancer Shown are the surface models for the 3D structures of HRAS (pdb# 1aa9), KRAS (pdb# 4epv) and NRAS (pdb# 3con; the only NRas 3D structure available in PDB, lacking residues 61-71), the three human RAS paralogs most frequently mutated in cancer. Mutations currently included in the TCGA catalog (Cancer Mut.) have been colored in green and DIRP in red. Overlapping positions (i.e., DIRP corresponding to residues mutated in cancer) are colored in yellow. For clarity, three rotating views are shown for each Ras protein.

DIRP specific positions constitute a large percentage (~ 38%) of the binding region of Ras with Guanine Exchange Factor (GEF) effectors (Table 2), such as SOS (Ras GEF), Epac2 (Rap GEF), RalGDS (Ras GEF) and the GTPase Activating Protein (GAP). The selected DIRP positions are also important for the tumor suppressor interaction regions in Ras recognized by selected antibodies (~35%) and, to a lesser extent, with the Ras Binding Domain (RBD) of different Ras triggered signal effectors (~19%), such as phosphoinositide 3-kinase, Raf, Byr2 or c-Raf1. In addition, several DIRPs map to residues frequently mutated in cancer (Figure 8 and Supplementary Table S6). This is particularly evident for residues such as G12, which together with G13 and Q61 account for 97% of RAS oncogenic mutations [21].

Other Ras complexes show a very low involvement of these DIRP specific positions in their Ras binding regions. For instance, the Ras binding region of the PLC epsilon Ras association domain only matches one position out of a total of 17 (Table 2). Results in this case suggest a low influence of the DIRP specific positions in the signal mediated by this domain. Only two complexes do not show any match to DIRP positions, the Ras complex with a mutated Raf protein and the interaction of Rheb (Ras like protein) with the PDEδ protein, a putative solubilizing factor for several prenylated Ras-subfamily proteins [22].

As mentioned, three DIRP positions (G77, E153 and C186) did not match any binding region, something that could be due to missing Ras complexes not yet registered in the PDB. Specifically, position 186 is known to be a conserved Cys residue located in a highly variable and unstructured carboxyl-terminal region of the Ras protein. Functionally, this residue is involved in the sorting and binding of Ras to the inner surface of the plasma membrane without which the protein is inactive [23–25]. The high degree of conservation in this position may suggest a co-localization of the DIRP in the inner membrane.

## DISCUSSION

In this work we carried out a comprehensive analysis of the relationship between the phylogeny of RAS proteins and their location in the interaction network. This was followed by sequence and structural analyses of DIRP conserved positions in the binding sites of RAS with its effectors. Our sequence analysis of these divergent but interacting proteins identified these key positions, which mapped to 3D binding regions in Ras that mediate the interaction with many of its effectors. These results support the idea that these conserved positions determine which DIRP lie close in the interactome, i.e sharing similar interaction contexts.

The prominent relationship of DIRP specific positions with Ras binding sites suggests that point mutations of these positions in somatic cells might result in rewiring of the Ras network, leading to pathological states [26], particularly for mutations that affect the on/off switch regulation. Mutations in Ras proteins can lead to a permanently activated cell proliferation state or an alteration of the Ras interaction network driving tumor development [27]. Furthermore, the change of just a couple of key residues between Ras and Ral paralogous proteins produces the interchange of specificity between their natural effectors [28, 29]. One of these interchanged residues is I36 of HRas, which corresponds to the DIRP specific position involved in the largest number of Ras complex binding sites (Table 3). Other DIRP positions match known tumor suppressor binding regions in Ras, suggesting that further investigation of DIRP positions could inspire novel anti-tumoral approaches. The methodology described in this work could be extended to the study of other protein families, applying the same pipeline.

The fact that many distant Ras paralogs share their context of interacting partners linked to the conservation of a few key positions supports the hypothesis of convergent evolution as highly probable in the Ras interaction network. Nevertheless, the phylogenetic model, observed in this work, shows that moving away in the Ras interactome involves the accumulation of many neutral and adaptive point mutations in a large process of sequence divergence, since there is no Ras paralogs close in the phylogenetic tree and distant in the interactome. However, it is also possible that the likelihood of convergent evolution increases when Ras sequences diverge. Certainly, the study of the potential role of the convergence evolution in shaping the Ras signaling network is a key topic that deserves a deeper phylogenetic analysis.

Despite intensive efforts in both basic and applied research in the field over the past 30 years, all attempts to develop an effective RAS inhibitor have consistently failed and thus RAS proteins have been historically considered undruggable [6, 21, 30]. Most studies have either tried to block RAS farnesylation to impair its translocation to the plasma membrane or to interfere with nucleotide binding, thus impairing RAS function. RAS farnesyltransferase inhibitors failed basically because cells can use alternative routes to add posttranslational modifications to RAS proteins. On the other hand, RAS GTPases bind nucleotides with picomolar affinities, what makes very difficult for an inhibitor to compete with the intracellular nucleotide pools, which are in the millimolar range [31]. More recently, however, several research groups have contributed with new 3D structures showing RAS GTPases in previously unknown conformations [32]. This set of data, together with new dynamic, computer-based models of RAS activation and a new methodology based on a combination of protein engineering and organic synthesis, i.e. chemical genetics [33, 34], have revealed transient pockets in the RAS proteins that can be targeted with small molecule inhibitors, thus leading to a renewed interest in RAS proteins as druggable targets [30]. Following computational modeling approaches, new molecules have been designed to inhibit RAS and RAL function. No inhibitors to RAP have been described to date. Two orthosteric peptides, HBS3 [35] and SAH-SOS1 [36], efficiently impair Ras-GEF interactions by mimetizing the αH helix of SOS1 positioned between the Ras switch I and switch II regions, involving residues L6, G15, L56, D57, E63, Y64, R73, T74 and Q99 in KRAS. Most of these residues are close (or are identical, e.g. Y64) to some DIRPs identified here. In addition, several groups have recently succeeded in the direct targeting of Ras-GEF interactions with small molecule inhibitors: by analyzing different RAS conformations, new druggable pockets were found involving residues K5, L6, V7, D54 (DIRP), I55, L56, Y71 (DIRP) and T74 [37, 38]. In addition, using NMR-based screen, Sun *et al*. identified a hydrophobic pocket located between the α2 helix of switch II (residues 60-70; amongst them: 60, 64, 66 and 68 are DIRP positions) and the central β sheet of KRas-G12D where to acommodate a collection of small molecules inhibitors, blocking interaction with its GEF Sos [39].

Inhibitors that impair RAL binding to its upstream GEFs have also been identified by structure-based virtual screening. Three compounds (RBC6, RBC8 and RCB10) able to interact with a GEF binding site, adjacent to switch II (residues 70-77) and the α2 helix (residues 78-85) of RALA, were identified by following that methodology [40]. By using molecular docking, the residues involved in the interaction were predicted to be those corresponding to positions T58, G60, R68, Y71 and M72 in HRAS, all of which (except M72) were identified as DIRP in our analysis. Interestingly, positions analogous to G10, A11 and Q95 in HRAS were predicted to mediate binding of the RBC inhibitors to RALA to impair interaction with GEFs and these three residues are close to other DIRP, i.e. the R103 and the catalytic G12. Thus, regardless their chemical nature (peptides or small molecules) the new set of inhibitory compounds designed to block protein-protein interactions in the Ras family network share a number of critical target residues that are identical to some DIRP conserved positions identified in our study.

In contrast to the prototypical RAS proteins, mutations in RAL or RAP proteins are infrequent and irrelevant in cancer (Supplementary Tables S6 and S7) [41]. However RALA and RALB are overexpressed in a number of tumors, most notably NSCLC and melanoma [9, 10, 42]. Thus, rewiring of the Ras network as a consequence of point mutations in DIRP residues is unlikely to occur because oncogenic mutations have only been found in HRAS, KRAS and NRAS, with G12 and Q61 accounting for the vast majority of hits (97% in HRAS, 99% in KRAS) (Supplementary Tables S6 and S7) [21]. On the other hand, rewiring due to changes in protein expression might occur in the context of RAL proteins, since altered expression of RAS isoforms is not a common feature in cancer (Supplementary Figure S1) although seems to relate with some RASopathies [21, 43]. However, the results presented here, i.e. the identification of DIRP conserved residues coincident with positions occupied by PPI inhibitors bound to RAS GTPases, suggest that these new group of inhibitors might not be as specific as initially expected. This is particularly important since we still lack information about their efficacy *in vivo*. The HBS3, SAH-SOS1 orthopeptides and the DCAI compound have been shown to reduce Ras-GTP levels and, in some instances, to inhibit ERK activation in cultured cells [35–37], but no *in vivo* experiments have been reported yet. On the contrary, the RBC8 and some related inhibitors of RAL-GEF interaction have been tested in mice xenografted with H2122 (lung) tumors, where they were able to reduce tumor growth in a dose-dependent manner [40]. Ras inhibitors may be used in combination with other ERK pathway inhibitors since, for instance, blocking MEK activity alone is not effective in the inhibition of Ras-dependent tumors. Also, oncogenic BRAF (V600E) inhibition can result in paradoxical activation of the pathway [30, 44, 45]. In any case, in light of the renewed interest in RAS GTPases as druggable targets in cancer [4], we believe that the identification of DIRP residues should be a valuable tool to assist in the evaluation of potential unspecificities of new Ras inhibitors.

The PPI networks used in this study are based on protein physical interactions collected from different data sources including *in vitro* experiments. These do not consider all the temporal or spatial regulation of gene expression (e.g. cellular compartmental barriers), which may prevent some interactions from occurring *in vivo*.

The results of this study add a novel perspective to the generally accepted model according to which phylogenetically close paralogous genes have similar interactions that diverge over time along with the divergency of their sequences [17–19]. Although the specificity of protein-protein interactions is the result of a complex combination of factors, our work suggests that a number of key positions are highly relevant to the interactions specificity. These positions could explain why divergent Ras proteins share close interaction contexts, increasing the probability of cross-talking amongst them. Finding compounds that target this functionally overlapping DIRP partners may help in the design of new treatment strategies.

## MATERIALS AND METHODS

### Phylogenetic trees of the Ras family

The phylogenetic trees for the 35 human Ras paralogous proteins used in this work were part of the dataset that was obtained in Diez *et al*. [2]. These original trees were the product of an exhaustive and accurate search for all the encoding genes in the Ras protein families across 24 eukaryotic species (putative pseudo-genes were excluded from the analysis). Ras human sequences were obtained from Uniprot and were aligned with their orthologs using ClustalW [46]. Finally, phylogenetic trees were constructed by Neighbor-Joining method implemented using the software Quicktree [47]. Tree topology reliability was assessed with the bootstrap method using 1000 replications.

### Protein-protein interaction networks data

The two protein-protein interaction networks used in this work were constructed using the following human datasets: PINA and STRING [48, 49]. STRING describes 263,666 interactions between 14,732 proteins from the integration of: BIND, DIP, GRID, HPRD, IntAct, MINT and PID databases [50–55]. PINA includes 108,477 unique interactions between 15,450 different proteins collected from six publicly available and manually curated databases: IntAct, MINT, BioGRID, DIP, HPRD and MIPS/MPact [56]. Only direct physical interactions were used in this study, avoiding both data derived from phylogenetic studies (preventing tautologies in the results when comparing with tree distances) and interactions obtained by textmining processes [57].

PINA covers 63% of the proteins present in the Ras phylogenetic tree and 31% of all possible connections between them, while STRING covers 77% and 52% respectively. Although the PPI data from PINA and STRING integrate a similar source of information (physical interactions, as mentioned), they show a different level of coverage of the Ras tree data and also a different network topology. Therefore, both were considered as valid and complementary datasets in this analysis.

### Pairwise distances in PPI networks and phylogenetic trees

RAS proteins were mapped onto the PPI networks and highly connected nodes (those with 300 or more connections) were removed, since these hubs introduce noise in distance calculations, as shown by Hériché *et al*. [58]. Out of the various algorithms tried, the Laplacian Exponential Diffusion Kernel (DK) and the Commute Time Kernel (CT) [58], were the ones that best fitted our purposes (see Section 1 in Supplementary material). Thus the pairwise protein distances within the networks were calculated using these methods. These are based on a calculation of the probability (*p*) of association of node pairs in the network using different statistical approaches for mathematically representing the network flow. Note that CT is also included as part of widely used tools such as GeneMANIA [59]. These probabilities were normalized and transformed into distances by calculating their negative natural logarithm (*–Ln(p)*) (Section 2 in Supplementary material). Statistical comparison between phylogenetic distances and PPI network matrices and their plot representations were performed using the computational software R [60].

Phylogenetic pairwise distances were calculated using the algorithm described by Pazos *et al*. [61], which uses protein tree files in the Newick Standard format as input and returns the numeric distance value for each pair. Later, scale corrections were carried out, applying an exponential mathematical transformation to the phylogenetic distances, so they could be plotted and compared together with the network distances (Figure 3 and Section 2 in Supplementary material).

### Selection of the divergent but interacting RAS pairs

To select divergent sequence pairs a maximum identity threshold of 45% was defined. This value was based on the BLOSUM 45 matrix [62], which was designed to weight amino acid substitutions between highly divergent sequences. This selected threshold correlated to a normalized phylogenetic distance between proteins greater than 1.7 (Figure 4).

To establish significant closeness between proteins in the interaction networks, a second threshold was set based on random distributions of the DK and CT network distance values. For each dataset and algorithm, this threshold was estimated accordingly to a statistical p-value = 0.05 (Supplementary Table S2 in Supplementary material).

Finally, those pairs with sequence identity ≤ 45% and DK and CT values ≥ DK_0.05_ and CT_0.05_, respectively, were used to select the final set of DIRP pairs (Table 1 and Figure 4, panel II).

### Multiple sequence alignment and measurement of amino acid conservation

A Multiple Sequence Alignment (MSA) of all Ras sequences was employed to assess amino acid conservation between protein pairs. This evaluation was done using the BLOSUM 45 amino acid substitution matrix to rate every change in each position of the sequences. The choice of BLOSUM 45 was based on the fact that this matrix was originally designed to compare highly divergent sequences with up to 45% identity, a condition that the dataset mostly fulfilled. Only those amino acids that aligned with the HRas sequence were used for the analysis of conservation. HRas was selected as a template for being the most studied protein in the family and one of the main pharmacological targets.

For each amino acid position in the MSA two values were calculated: i) the average level of conservation between DIRP (positive control) based on binary alignments of all pairs in the DIRP dataset and ii) the average level of conservation of an equal number of randomly selected Ras protein pairs (negative control) using the same approach as in “i)”. These two values were then normalized to the average level of conservation of the global MSA. Based on the random model results, a p-value was calculated and used as a threshold to select the significantly conserved amino acids (Figure 5 and Figure 6).

Visualization and edition of the MSA was done using the software JalView V2.7 [63]. A general pipeline of the process can be seen in steps A, B and C in Figure 5.

### Random models

For each PPI network and algorithm used, random models were generated at different stages of the work in order to estimate the statistical significance of the results (i.e. to be used as negative controls).

### Random models of the interactome

A hundred PPI network models were built for every PPI network used, randomly permuting the partners of each node while maintaining their degree of connection. Network distances were then calculated in these models and compared to phylogenetic distances.

### Random set of aligned protein pairs

A hundred sets of protein pairs were built by randomly selecting aligned pairs out of the MSA. Random set sizes were kept the same as the original dataset (see Table 1 for information about the number of aligned pairs in each case).

### Acquisition and processing of Ras complexes structural data

All known interaction complexes of human Ras proteins were downloaded from the Protein Data Bank (PDB) [20]. Those with 100% sequence identity were grouped together (Supplementary Table S3 in Supplementary material) and then clustered into functional categories according to their 3D structural similarity (rms < 1.0; Supplementary Table S4 in Supplementary material). For each functional group, the Ras interaction surface was determined by computing the difference in the solvent accessible surface area of Ras amino acids between the complex and unbounded states, using the DSSP software [64]. Data regarding mutation frequencies were obtained from COSMIC (http://cancer.sanger.ac.uk/cosmic) [5] Structural models were rendered with The PyMOL Molecular Graphics System, Version 1.8 Schrödinger, LLC.

## SUPPLEMENTARY DATA








