# Supplementary material for: Exploring the interactions of the RAS family in the human protein network and their potential implications in RAS-directed therapies
Source: Oncotarget. 2016 Oct 3;7(46):75810–26. doi: 10.18632/oncotarget.12416 (PMC5342780; doi:10.18632/oncotarget.12416)
Supplement: Supplementary file 1 [file oncotarget-07-75810-s001.pdf]

Exploring the interactions of the RAS family in the human protein network and their potential implications in RAS-directed therapies

SUPPLEMENTARY DATA

Pairwise distance measures in protein-protein interaction networks

There are many methods to calculate distance or similarity between nodes in a network. The simplest one is to calculate the shortest path between each pair. This can be done using methods such as: Dijkstra, A* or Floyd-Warshall [65-67]. These methods have some disadvantages: the distances are discrete so we cannot make a fine-tuning and the topology of the analyzed network is not taken into account. For example, node 3 and node 8 in Figure I have the same direct distance as node 8 and node 11. However, the significance of those relationships is very different due to the cluster distribution context derived from the topology of the network. To avoid such a problem, different probabilistic based algorithms can be applied to each protein-protein interaction network to obtain measures of distance between nodes that do take into account the biological context behind a topological conformation, such as Diffusion Kernels [68]: Laplacian Exponential Diffusion Kernel (DK) or Commute Time Diffusion Kernel (CT).

As it was mentioned, probabilistic methods take into account the effects of network topology on the distance measurement between nodes as it can be seen in Figure II. The short path measure is the same for the three examples shown in that Figure, however the probabilistic algorithm is able to distinguish between the three given situations, and gives more weight to the x and y relationship in C than in A or B.

Laplacian exponential diffusion kernel (DK)

This method belongs to the group of *Diffusion Kernel* methods, that are mathematical approximations for measuring network flow and network topology between two given nodes based on the calculation of all possible paths between them, in both directions, and calculating an average in a matrix based form. A first operation must be done: $L=D-A$. Where A denotes the adjacency matrix of the graph, D is the diagonal degree matrix and as a result, L is the Laplacian matrix.

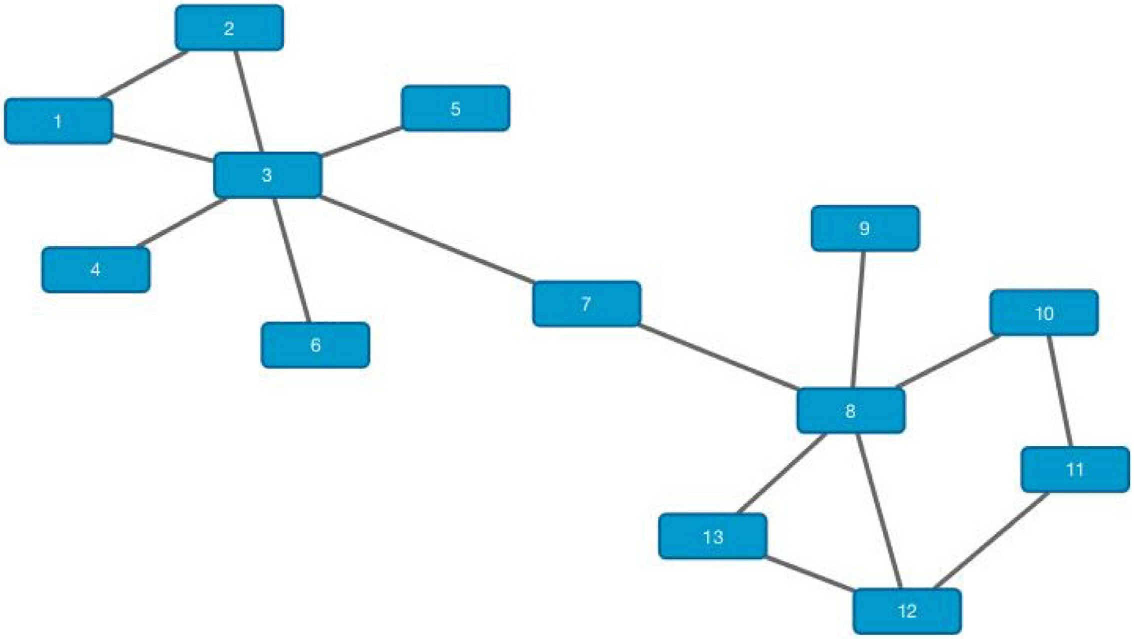

Figure I: Example of a protein interaction subnetwork. Proteins -represented by boxes- are the nodes of the network while the edges represent the interactions between them.

To obtain the final Laplacian Exponential Diffusion Kernel matrix the next formula must be applied: $K = \exp(-\beta L)$. The value of β was established to 0.02, as an optimization of the associated Receiver Operating Characteristic curve within a cross-validation procedure.

The final result is an $n \times n$ symmetric matrix (being n the number of nodes in the network) containing the distances between each pair of proteins.

Commute time diffusion kernel (CT)

The second method used in this study is also a subtype of *Diffusion Kernel*. In this case, the algorithm is based on a mathematical computation that estimates the average number of steps a random walker in the network needs to go from one node to another and back [70].

The formula is: $K = L^{+}$, where L is the Laplacian matrix, obtained as in the previous method, and L^{+} is the pseudo-inverse of L.

Distance measures normalization for comparison

Due to the intrinsic nature of the data obtained from CT and DK algorithms (non-linear transformations), their values are exponentially distributed (panel A in Figure III). Conversely, phylogenetic distances distribution, seem to have a logarithmic behavior (panel B in Figure III). A proper mathematical treatment is required in both measures in order to be compared. According to that, a normalization was carried out by applying exponential and logarithmical filters to the network and tree values respectively (Figure IV).

As it can be seen in Figure IV, a raw comparison between both measures without any kind of adjustment it would be hard to read because of their different distributions, therefore we carried out the previously mentioned normalization process, after which we were able to analyze correctly the relationship between both measures.

Direct interacting proteins of four ras pairs from the dirp dataset

All interacting proteins (Ras and their direct interacting partners) are shown with their ENSMBL ID, gene symbol and functional/gene description.

RHEB and RAP1A:

- ENSP00000262187: RHEB - Ras homolog enriched in brain; Stimulates the phosphorylation of S6K1 and EIF4EBP1 through activation of mTORC1 signaling. Activates the protein kinase activity of mTORC1. Has low intrinsic GTPase activity.

- ENSP00000348786: RAP1A - RAP1A, member of RAS oncogene family; Induces morphological reversion of a cell line transformed by a Ras oncogene. Counteracts the mitogenic function of Ras, at least partly because it can interact with Ras GAPs and RAF in a competitive manner.

- ENSP00000219476: TSC2 - tuberous sclerosis 2; In complex with TSC1, inhibits the nutrient-mediated or growth factor-stimulated phosphorylation of S6K1 and EIF4EBP1 by negatively regulating mTORC1 signaling. Acts as a GTPase- activating protein (GAP) for the small GTPase RHEB, a direct activator of the protein kinase activity of mTORC1. Implicated as a tumor suppressor. Involved in microtubule-mediated protein transport, but this seems to be due to unregulated mTOR signaling. Stimulates weakly the intrinsic GTPase activity of the Ras-related proteins RAP1A and RAB5 *in vitro*.

- ENSP00000251849: RAF1 - v-raf-1 murine leukemia viral oncogene homolog 1; Serine/threonine-protein kinase that acts as a regulatory link between the membrane-associated Ras GTPases and the MAPK/ERK cascade, and this critical regulatory link functions as a switch determining cell fate decisions including proliferation, differentiation, apoptosis, survival and oncogenic transformation. RAF1 activation initiates a mitogen-activated protein kinase (MAPK) cascade that comprises a sequential phosphorylation of the dual-specific MAPK kinases (MAP2K1/MEK1 and MAP2K2/MEK2).

- ENSP00000287600: PDE6D - phosphodiesterase 6D, cGMP-specific, rod, delta; Acts as a GTP specific dissociation inhibitor (GDI). Increases the affinity of ARL3 for GTP by several orders of magnitude and does so by decreasing the nucleotide dissociation rate. Stabilizes Arl3-GTP by decreasing the nucleotide dissociation (By similarity).

- ENSP00000288602: BRAF - v-raf murine sarcoma viral oncogene homolog B1.

- ENSP00000354558: MTOR - mechanistic target of rapamycin (serine/threonine kinase); Serine/threonine protein kinase which is a central regulator of cellular metabolism, growth and survival in response to hormones, growth factors, nutrients, energy and stress signals. Functions as part of 2 structurally and functionally distinct signaling complexes mTORC1 and mTORC2 (mTOR complex 1 and 2). Activated mTORC1 up-regulates protein synthesis by phosphorylating key regulators of mRNA translation and ribosome synthesis.

MRAS and RAP2A:

- ENSP00000289104: MRAS - muscle RAS oncogene homolog; May serve as an important signal transducer for a novel upstream stimuli in controlling cell proliferation. Weakly activates the MAP kinase pathway.

- ENSP00000245304: RAP2A - RAP2A, member of RAS oncogene family; Small GTP-binding protein which

cycles between a GDP- bound inactive and a GTP-bound active form. In its active form interacts with and regulates several effectors including MAP4K4, MINK1 and TNIK. Part of a signaling complex composed of NEDD4, RAP2A and TNIK which regulates neuronal dendrite extension and arborization during development. More generally, it is part of several signaling cascades and may regulate cytoskeletal rearrangements, cell migration, cell adhesion and cell spreading.

- ENSP00000222145: RASIP1 - Ras interacting protein 1; Required for the proper formation of vascular structures that develop via both vasculogenesis and angiogenesis. Acts as a critical and vascular-specific regulator of GTPase signaling, cell architecture,

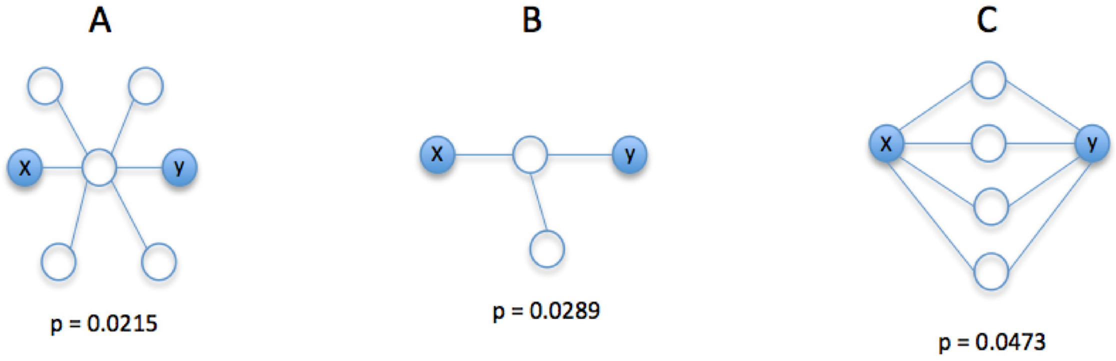

Figure II: Example of different network topologies connecting two proteins (x and y). The computed CT probability score (p) is shown below each network model. **A.** Highly connected node (hub) between *x* and *y*. **B.** protein *x* and protein *y* are connected through a protein that has a small number of interactions. **C.** Protein *x* and protein *y* are connected by several paths. Adapted from Kohler *et al*. [69].

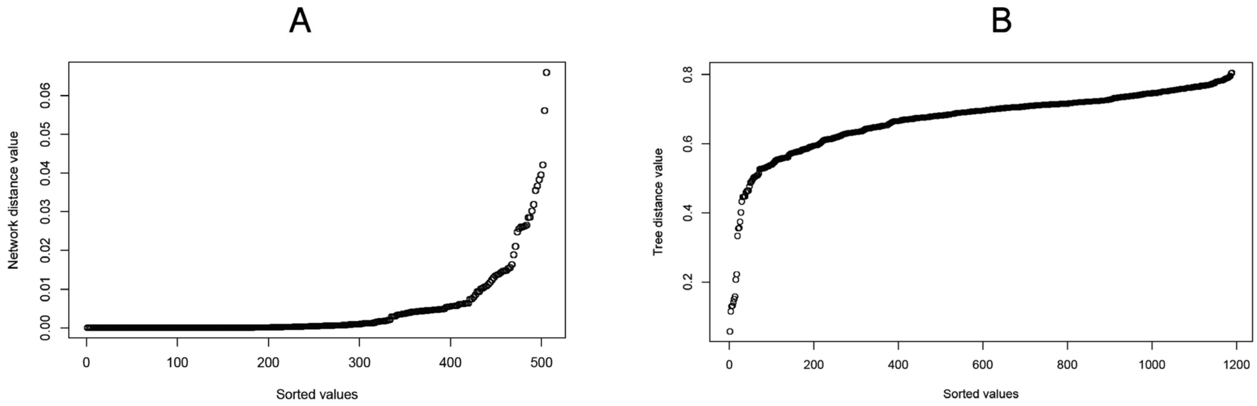

Figure III: Example of network distance values distribution and phylogenetic similarity values distribution. A. Pairwise network (CT) distances of the human Ras proteins family in the STRING Experimental dataset. **B.** Pairwise phylogenetic distances distribution of the same proteins.

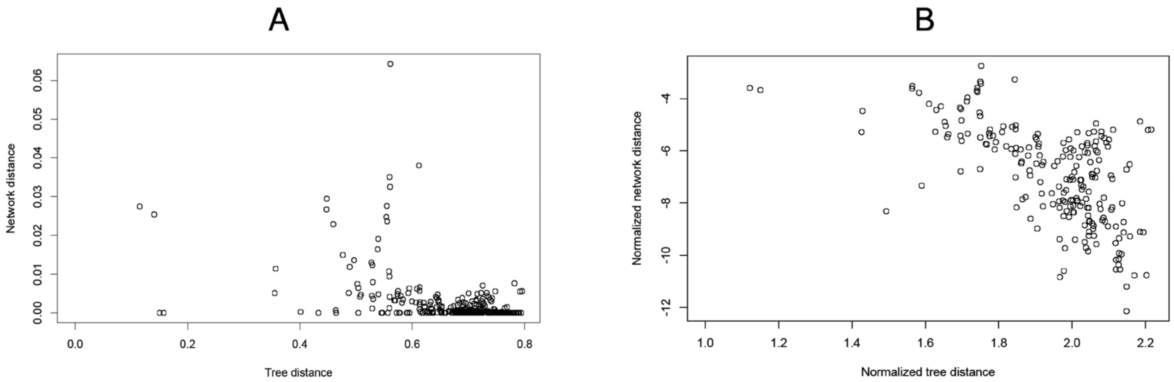

Figure IV: Effect of the normalization of measures when comparing network distance and phylogenetic distance. Pairwise phylogenetic distance (x-axis) versus pairwise network distance (y-axis) before **A.** and after **B.** the normalization.

and adhesion, which is essential for endothelial cell morphogenesis and blood vessel tubulogenesis. Regulates the activity of Rho GTPases in part by recruiting ARHGAP29 and suppressing RhoA signaling and dampening ROCK and MYH9 activities in endothelial cells (By similarity). May act as effector for Golgi-bound HRAS and other Ras-like proteins.

- ENSP00000251849: RAF1 - v-raf-1 murine leukemia viral oncogene homolog 1; Serine/threonine-protein kinase that acts as a regulatory link between the membrane-associated Ras GTPases and the MAPK/ERK cascade, and this critical regulatory link functions as a switch determining cell fate decisions including proliferation, differentiation, apoptosis, survival and oncogenic transformation. RAF1 activation initiates a mitogen-activated protein kinase (MAPK) cascade that comprises a sequential phosphorylation of the dual-specific MAPK kinases (MAP2K1/MEK1 and MAP2K2/MEK2).

- ENSP00000296859: RAPGEF6 - Rap guanine nucleotide exchange factor (GEF) 6; Guanine nucleotide exchange factor (GEF) for Rap1A, Rap2A and M-Ras GTPases. Does not interact with cAMP.

- ENSP00000343656: RAPGEF5 - Rap guanine nucleotide exchange factor (GEF) 5; Guanine nucleotide exchange factor (GEF) for RAP1A, RAP2A and MRAS/M-Ras-GTP. Its association with MRAS inhibits Rap1 activation.

- ENSP00000347443: RASSF5 - Ras association (RalGDS/AF-6) domain family member 5.

- ENSP00000361120: RALGDS - ral guanine nucleotide dissociation stimulator; Stimulates the dissociation of GDP from the Ras-related RalA and RalB GTPases which allows GTP binding and activation of the GTPases. Interacts and acts as an effector molecule for R-Ras, H-Ras, K-Ras, and Rap.

- ENSP00000383623: MLLT4 - myeloid/lymphoid or mixed-lineage leukemia (trithorax homolog, Drosophila).

RASD2 and KRAS:

- ENSP00000216127: RASD2 - RASD family, member 2; GTPase signaling protein that binds to and hydrolyzes GTP. Regulates signaling pathways involving G-proteins-coupled receptor and heterotrimeric proteins such as GNB1, GNB2 and GNB3. May be involved in selected striatal competencies, mainly locomotor activity and motor coordination

- ENSP00000256078: KRAS - v-Ki-ras2 Kirsten rat sarcoma viral oncogene homolog; Ras proteins bind GDP/GTP and possess intrinsic GTPase activity

- ENSP00000251849: RAF1 - v-raf-1 murine leukemia viral oncogene homolog 1; Serine/threonine-protein kinase that acts as a regulatory link between the membrane-associated Ras GTPases and the MAPK/ERK cascade, and this critical regulatory link functions as a switch determining cell fate decisions including proliferation, differentiation, apoptosis, survival and oncogenic transformation. RAF1 activation initiates a mitogen-activated protein kinase (MAPK) cascade that comprises a sequential phosphorylation of the dual-specific MAPK kinases (MAP2K1/MEK1 and MAP2K2/MEK2) and the extracellular signal-regulated kinases.

- ENSP00000263967: PIK3CA - phosphatidylinositol-4,5-bisphosphate 3-kinase, catalytic subunit alpha; Phosphoinositide-3-kinase (PI3K) that phosphorylates PtdIns (Phosphatidylinositol), PtdIns4P (Phosphatidylinositol 4- phosphate) and PtdIns(4,5)P2 (Phosphatidylinositol 4,5- bisphosphate) to generate phosphatidylinositol 3,4,5-trisphosphate (PIP3). PIP3 plays a key role by recruiting PH domain-containing proteins to the membrane, including AKT1 and PDPK1, activating signaling cascades involved in cell growth, survival, proliferation, motility and morphology.

RIT2 and RRAS:

- ENSP00000321805: RIT2 - Ras-like without CAAX 2; Binds and exchanges GTP and GDP (By similarity).

- ENSP00000246792: RRAS - related RAS viral (r-ras) oncogene homolog; Regulates the organization of the actin cytoskeleton.

- ENSP00000339007: GRB2 - growth factor receptor-bound protein 2; Adapter protein that provides a critical link between cell surface growth factor receptors and the Ras signaling pathway.

- ENSP00000361120: RALGDS - ral guanine nucleotide dissociation stimulator; Stimulates the dissociation of GDP from the Ras-related RalA and RalB GTPases which allows GTP binding and activation of the GTPases. Interacts and acts as an effector molecule for R-Ras, H-Ras, K-Ras, and Rap.

- ENSP00000383623: MLLT4 - myeloid/lymphoid or mixed-lineage leukemia (trithorax homolog, Drosophila); translocated to, 4.

- ENSP00000384675: SOS1 - son of sevenless homolog 1 (Drosophila); Promotes the exchange of Ras-bound GDP by GTP. Catalytic component of a trimeric complex that participates in transduction of signals from Ras to Rac by promoting the Rac-specific guanine nucleotide exchange factor (GEF) activity (By similarity).

A

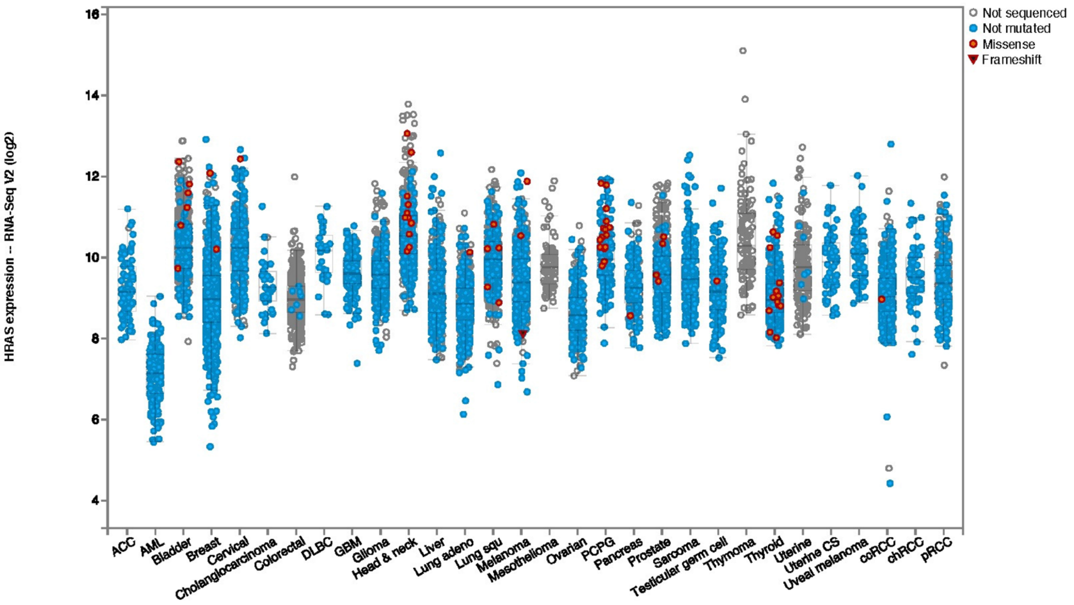

B

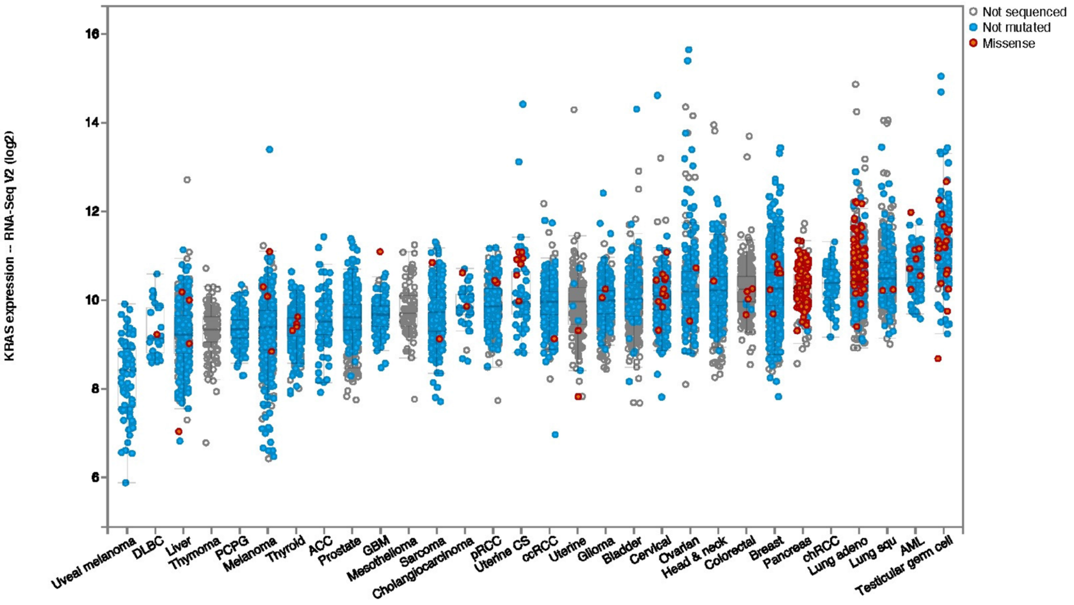

(*Continued*)

C

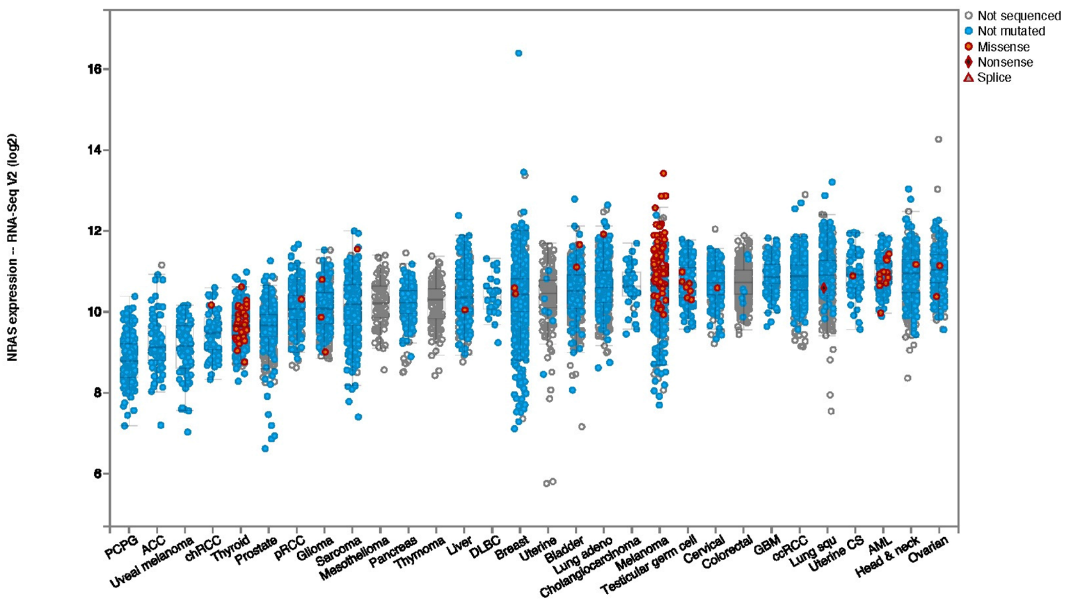

Supplementary Figure S1: Expression of human HRAS, KRAS and NRAS genes in cancer. A-C. Data was obtained from the TCGA database through the cBioPortal web module and mostly represent quantitative transcriptome analysis using RNA-Seq. Data obtained using other quantitative techniques (i.e. microarrays) are labeled as gray dots. Values are shown in a log2 scale. For each type of cancer, those samples containing wild-type versions of the genes are labeled in blue while those containing mutated versions are labeled in red.

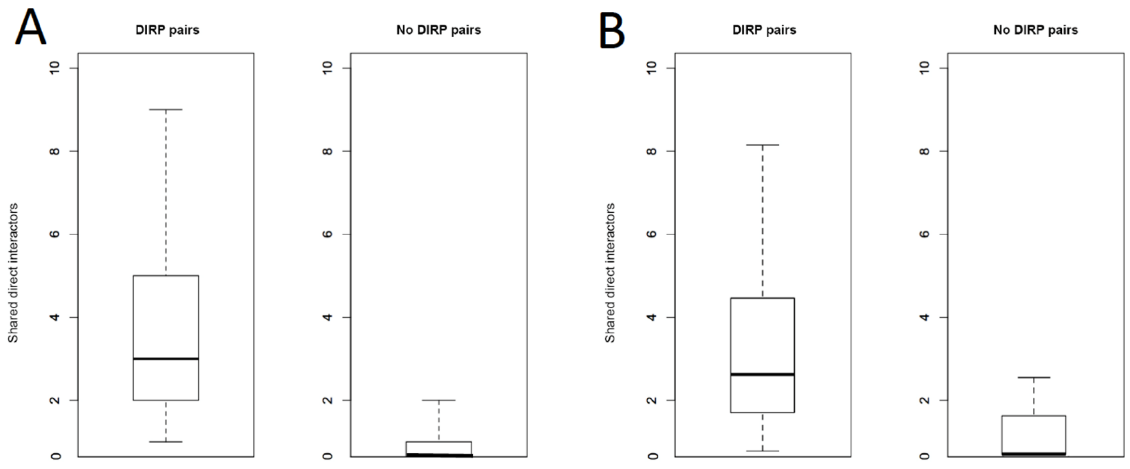

Supplementary Figure S2: Distribution of number of shared direct interacting proteins between Ras paralogs in the DIPR and the No-DIRP datasets, based on A. Commute Time –CT- kernel and **B.** Difussion Kernel –DK- metrics. All proteins with direct physical interaction, based on experimental evidences in the STRING database, were retrieved for all the Ras pairs in the DIRP dataset, and for an equal number of randomly selected No-DIRP Ras protein pairs (negative control). The distributions median values are indicated with thick lines. All the direct interacting partners for each DIRP pair are shown in a supplemental plain text file.

Supplementary Table S1: Selected DIRP specific positions

See Supplementary File 1

Supplementary Table S2: Boundaries for significant network distances (network closeness threshold)

PPI network dataset / Normalized network distance boundaries	$CT_{0.05}$*	$DK_{0.05}$*
**PINA**	-6.5	-12
**STRING EXP**	-6.5	-11.5

*$CT_{0.05}$ and $DK_{0.05}$ columns represent CT and DK network distance cut off boundary values (log normalized measure) for a p-value = 0.05.

Supplementary Table S3: List of known complexes in PDB for each of the Ras human paralogous proteins

See Supplementary File 1

Supplementary Table S4: Ras 3D complexes clustering based on their structural similarity

See Supplementary File 1

Supplementary Table S5: No-published directly shared interacting proteins between the four selected example of Ras DIRP pairs

Ras DIRP pair	No published interaction	Experimental Evidence	Experiment annotation Source	Association in Curated Databases	Co-Mentioned in PubMed Abstracts (score)
RHEB/RAP1A	RHEB /PDE6D	Fluorescent resonance energy transfer assay; Two-hybrid assay; Fluorescence polarization spectroscopy assays; and pull down assay	DIP<br>GRID<br>INTACT	NONE	0.043
RAP2A /MRAS	MRAS / RASSF5	*In vitro* and *in vivo* assays	HPRD	NONE	0.070
RAP2A /MRAS	MRAS /RASIP1	*In vitro* assay	HPRD	NONE	0.045
RAP2A /MRAS	RAP2A/ RASSF5	Detected by *in vitro* and yeast 2-hybrid assays	HPRD	NONE	NONE
RASD2/KRAS	RASD2/RAF1	Detected by Reconstituted Complex assay	BioGRID	NONE	NONE
RASD2/KRAS	RASD2/ PIK3CA	Detected by Reconstituted Complex assay	BioGRID	NONE	NONE
RIT2/RRAS	RIT2/ GRB2	*Detected by psi-mi:"MI:0081"(peptide array) assay*	IntAct	NONE	0.043

All Ras-partner interactions were systematically searched in the whole PubMed literature database and other functionally curated databases, using the STRING text-mining web tools (http://string-db.org). An interaction was considered no-published when no functional study was linked to the interaction beyond the simple experimental evidence. STRING gives a score between 0 and 1, being 1 the highest confidence value. We selected as no-published those human protein interactions with no association in any functionally curated database and with a score below 0.1 in the STRING PubMed text-mining tool.

Supplementary Table S6: Catalogue of all cancer-related mutations located in the 35 Ras human paralogs. Data was retrieved from the COSMIC public database. In every worksheet, column C (in yellow) contains the list of amino-acid changes. Red tabs have been used to facilitate location of the mutational data for *KRAS*, *NRAS* and *HRAS*

See Supplementary File 2

Supplementary Table S7: Incidence of Ras paralogs' mutations in human tumors. Data was retrieved from the COSMIC public database

See Supplementary File 3
