# Supplementary material for: Exploring the interactions of the RAS family in the human protein network and their potential implications in RAS-directed therapies
Source: Oncotarget. 2016 Oct 3;7(46):75810–26. doi: 10.18632/oncotarget.12416 (PMC5342780; doi:10.18632/oncotarget.12416)
Supplement: Supplementary file 4 [file oncotarget-07-75810-s004.docx]

Table S1. Selected DIRP specific positions.

| **Position** | **String Exp CT** | **String Exp DK** | **PINA CT** | **PINA DK** |
| --- | --- | --- | --- | --- |
| G12 |  |  | 3.13 |  |
| T20 |  |  | 2.49 |  |
| Q22 | 2.85 | 2.46 | 3.41 | 2.61 |
| F28 | 3.54 | 2.60 | 3.73 | 2.61 |
| Y32 | 2.86 |  | 2.15 |  |
| D33 | 2.56 |  | 3.46 | 3.42 |
| P34 | 3.58 | 3.24 | 3.57 | 2.39 |
| T35 | 2.29 | 2.46 | 2.28 |  |
| I36 | 2.40 |  | 2.38 |  |
| D54 | 2.36 |  | 2.55 |  |
| T58 | 2.52 |  | 2.33 |  |
| A59 | 2.86 | 2.28 | 3.56 | 3.09 |
| G60 | 3.54 | 3.21 | 4.05 | 2.90 |
| Y64 | 2.30 |  | 3.50 |  |
| A66 |  |  | 2.12 |  |
| R68 |  |  | 3.10 |  |
| Y71 | 3.72 |  | 4.63 | 3.73 |
| G77 | 2.00 |  | 3.45 | 2.51 |
| V103 |  |  | 2.24 |  |
| I139 | -3.62 | -3.16 | -3.01 | -3.47 |
| E153 | 2.14 |  | 2.33 |  |
| C186 | 3.50 | 4.58 | 2.86 |  |

Amino acid positions using HRAS sequence as reference. The differential conservation values for each position are shown in columns 2 to 4, for each metric used and each network studied.

Table S3. List of known complexes in PDB for each of the Ras human paralogous proteins.

| Protein | Ensembl protein ID | UniProt protein ID | PDB entries | PDB known complexes |
| --- | --- | --- | --- | --- |
| RAL. v-ral simian leukemia viral oncogene homolog A (ras related). | **ENSP00000005257** | **P11233** | **1UAD;1ZC3;1ZC4;2A78;2A9K;2BOV** | **1UAD;1ZC3;1ZC4;2A78;2A9K;2BOV** |
| **REM. RAS (RAD and GEM)-like GTP-binding 1.** | **ENSP00000201979** | **O75628** | **2NZJ** |  |
| **RASL10A. RAS-like, family 10, member A; Potent inhibitor of cellular proliferation.** | **ENSP00000216101** | **Q92737** |  |  |
| **RASD2. RASD family, member 2; GTPase signaling protein that binds to and hydrolyzes GTP.** | **ENSP00000216127** | **Q96D21** |  |  |
| **RASL12. RAS-like, family 12.** | **ENSP00000220062** | **Q9NYN1** | **3C5C** |  |
| **RASD1. RAS, dexamethasone-induced 1; Small GTPase.** | **ENSP00000225688** | **Q9Y272** |  |  |
| **RASL11A. RAS-like, family 11, member A; Regulator of rDNA transcription.** | **ENSP00000241463** | **Q6T310** |  |  |
| **RAP2A. RAP2A, member of RAS oncogene family; Small GTP-binding protein which cycles between a GDP- bound inactive and a GTP-bound active form.** | **ENSP00000245304** | **P10114** | **1KAO;2RAP;3RAP** |  |
| **RRAS. Related RAS viral (r-ras) oncogene homolog; Regulates the organization of the actin cytoskeleton.** | **ENSP00000246792** | **P10301** | **2FN4** |  |
| **RASL11B. RAS-like, family 11, member B** | **ENSP00000248706** | **Q9BPW5** |  |  |
| **KRAS. v-Ki-ras2 Kirsten rat sarcoma viral oncogene homolog** | **ENSP00000256078** | **P01116** | **1D8D;1D8E;1KZO;1KZP;3GFT;4DSN;4DSO;4EPR;4EPT;4EPV;4EPW;4EPX;4EPY** |  |
| **RRAS2. related RAS viral (r-ras) oncogene homolog 2; It is a plasma membrane-associated GTP-binding protein with GTPase activity.** | **ENSP00000256196** | **P62070** | **2ERY** |  |
| **RERG. RAS-like, estrogen-regulated, growth inhibitor; Binds GDP/GTP and possesses intrinsic GTPase activity.** | **ENSP00000256953** | **Q96A58** | **2ATV** |  |
| **RHEB. Ras homolog enriched in brain; Stimulates the phosphorylation of S6K1 and EIF4EBP1 through activation of mTORC1 signaling.** | **ENSP00000262187** | **Q15382** | **1XTQ;1XTR;1XTS;3SEA;3T5G** | **3T5G** |
| **REM2. RAS (RAD and GEM)-like GTP binding 2; Binds GTP saturably and exhibits a low intrinsic rate of GTP hydrolysis.** | **ENSP00000267396** | **Q8IYK8** | **3CBQ** |  |
| **RASL10B. RAS-like, family 10, member B.** | **ENSP00000268864** | **Q96S79** |  |  |
| **MRAS. Muscle RAS oncogene homolog; May serve as an important signal transducer for a novel upstream stimuli in controlling cell proliferation.** | **ENSP00000289104** | **O14807** |  |  |
| **GEM. GTP binding protein overexpressed in skeletal muscle.** | **ENSP00000297596** | **P55040** | **2CJW;2G3Y;2HT6** | **2CJW (false complex)** |
| **RRAD. Ras-related associated with diabetes.** | **ENSP00000299759** | **P55042** | **2DPX;2GJS;3Q72;3Q7P;3Q7Q** |  |
| **RHEBL1. Ras homolog enriched in brain like 1; Binds GTP and exhibits intrinsic GTPase activity.** | **ENSP00000301068** | **Q8TAI7** | **3OES** |  |
| **KBRAS2. NFKB inhibitor interacting Ras-like 2.** | **ENSP00000312773** | **H7BXP1** |  |  |
| **RAP2B. Member of RAS oncogene family; Small GTP-binding protein which cycles between a GDP- bound inactive and a GTP-bound active form.** | **ENSP00000319096** | **P61225** |  |  |
| **RIT2. Ras-like without CAAX 2; Binds and exchanges GTP and GDP.** | **ENSP00000321805** | **Q99578** |  |  |
| **Di-Ras1. DIRAS family, GTP-binding RAS-like 1.** | **ENSP00000325836** | **O95057** | **2GF0** |  |
| **ERAS. ES cell expressed Ras; Ras proteins bind GDP/GTP and possess intrinsic GTPase activity.** | **ENSP00000339136** | **Q7Z444** |  |  |
| **RAP2C, member of RAS oncogene family** | **ENSP00000340274** | **Q9Y3L5** |  |  |
| **RAP1A. Member of RAS oncogene family; Induces morphological reversion of a cell line transformed by a Ras oncogene.** | **ENSP00000348786** | **P62834** | **1C1Y;1GUA;3KUC** | **1C1Y;1GUA;3KUC** |
| **RIT. Ras-like without CAAX 1.** | **ENSP00000357305** | **Q5VY89** |  |  |
| **NRAS. Neuroblastoma RAS viral (v-ras) oncogene homolog.** | **ENSP00000358548** | **P01111** | **3CON** |  |
| **DIRAS2. DIRAS family, GTP-binding RAS-like 2; Displays low GTPase activity and exist predominantly in the GTP-bound form.** | **ENSP00000364919** | **Q96HU8** | **2ERX** |  |
| **NKIRAS1. NF-kappa-B inhibitor-interacting Ras-like protein 1.** | **ENSP00000373411** | **Q9NYS0** |  |  |
| **RAP1B. Member of RAS oncogene family.** | **ENSP00000377085** | **P61224** | **3BRW;3CF6;4DXA** | **3CF6;4DXA** |
| **DIRAS3. GTP-binding RAS-like 3** | **ENSP00000378627** | **O95661** |  |  |
| **HRAS1. Harvey rat sarcoma viral oncogene homolog.** | **ENSP00000380723** | **P01112** | **121P;1AA9;1AGP;1BKD;1CLU;1CRP;1CRQ;1CRR;1CTQ;1GNP;1GNQ;1GNR;1HE8;1IAQ;1IOZ;1JAH;1JAI;1K8R;1LF0;1LF5;1LFD;1NVU;1NVV;1NVW;1NVX;1P2S;1P2T;1P2U;1P2V;1PLJ;1PLK;1PLL;1Q21;1QRA;1RVD;1WQ1;1XCM;1XD2;1XJ0;1ZVQ;1ZW6;221P;2C5L;2CE2;2CL0;2CL6;2CL7;2CLC;2CLD;2EVW;2GDP;2LCF;2Q21;2QUZ;2RGA;2RGB;2RGC;2RGD;2RGE;2RGG;2UZI;2VH5;2X1V;3DDC;3I3S;3K8Y;3K9L;3K9N;3KKM;3KKN;3KUD;3L8Y;3L8Z;3LBH;3LBI;3LBN;3LO5;3OIU;3OIV;3OIW;3RRY;3RRZ;3RS0;3RS2;3RS3;3RS4;3RS5;3RS7;3RSL;3RSO;3TGP;421P;4DLR;4DLS;4DLT;4DLU;4DLV;4DLW;4DLX;4DLY;4DLZ;4DST;4DSU;4EFL;4EFM;4EFN;4Q21;521P;5P21;621P;6Q21;721P;821P** | **1BKD;1HE8;1K8R;1LFD;1NVU;1NVV;1NVW;1NVX;1WQ1;1XD2;2C5L;2UZI;2VH5;3DDC;3KUD;6Q21** |
| **RALB. Ras-related protein.** | **ENSP00000384328** | **Q6ZS74** |  |  |

From left to right: Protein description. Protein Id in ENSEMBL database. Protein accession number in Uniprot database. PDB Ids of the 3D structures containing each Ras paralog. PDB Ids of the 3D structures containing each Ras paralog in complex with other proteins.

Table S4. Ras 3D complexes clustering based on their structural similarity.

| Protein | Ensembl protein ID | Cluster ID | Complexes in cluster |
| --- | --- | --- | --- |
| **HRAS1. Harvey rat sarcoma viral oncogene homolog.** | **ENSP00000380723** | **3DDC** | **3DDC** |
|  |  | **2C5L** | **2C5L / 2UZI / 2VH5 / 1HE8** |
|  |  | **1NVU** | **1NVU / 1NVX** |
|  |  | **1LFD** | **1LFD** |
|  |  | **6Q21** | **6Q21 / 3KUD / 1XD2 / 1WQ1 / 1NVW / 1NVV / 1K8R / 1BKD** |
| **RAP1B. Member of RAS oncogene family.** | **ENSP00000377085** | **4DXA** | **4DXA** |
|  |  | **3CF6** | **3CF6** |
| **RAP1A. Member of RAS oncogene family; Induces morphological reversion of a cell line transformed by a Ras oncogene.** | **ENSP00000348786** | **1C1Y** | **1C1Y** |
|  |  | **3KUC** | **3KUC / 1GUA** |
| **RHEB. Ras homolog enriched in brain; Stimulates the phosphorylation of S6K1 and EIF4EBP1 through activation of mTORC1 signaling.** | **ENSP00000262187** | **3T5G** | **3T5G** |
| RAL. v-ral simian leukemia viral oncogene homolog A (ras related). | **ENSP00000005257** | **1ZC3** | **1ZC3 / 1ZC4** |
|  |  | **2A9K** | **2A9K / 1UAD / 2A78** |
|  |  | **2BOV** | **2BOV** |

Clusterization of Ras 3D complexes with an rmsd ≤ 1.0 (root mean square deviation). From left to right: Protein description. Protein Ids in ENSEMBL database. PDB Ids of the 3D structures taken as the general representative Id for each cluster. PDB Ids of the 3D structures that are included in each cluster.
